# Necrotizing hepatitis caused by *Clostridium novyi* type B in a dog with no predisposing liver lesions: a case report

**DOI:** 10.1186/s12917-022-03436-9

**Published:** 2022-10-05

**Authors:** Brie Trusiano, S. Michelle Todd, Sarah Barrett, Michael Ciepluch, Alexandra Fox, Diamond McClendon, Kevin K. Lahmers, Vanessa J. Oakes, Francisco Carvallo, Virginia Corrigan, Tessa E. LeCuyer

**Affiliations:** 1 Department of Biomedical Sciences and Pathobiology, Virginia-Maryland College of Veterinary Medicine, VA Tech, 205 Duck Pond Drive, Blacksburg, VA 24061 USA; 2 Virginia Tech Animal Laboratory Services, VA-Maryland College of Veterinary Medicine, Virginia Tech, Blacksburg, VA 24061 USA; 3 Present Address: Zoetis, Inc., US NJ Remote, Parsippany-Troy Hills, USA; 4 Department of Small Animal Clinical Sciences, Virginia-Maryland College of Veterinary Medicine, Virginia Tech, Blacksburg, VA 24061 USA; 5 Present Address: Department of Rural Resilience and Innovation, Appalachian State University, Boone, NC 28608 USA

**Keywords:** Infectious necrotic hepatitis, *Clostridium novyi*, Canine infectious hepatitis, Case report

## Abstract

**Background:**

Infectious necrotic hepatitis (INH) is typically a disease of ruminants caused by *Clostridium novyi* type B. Growth of the causative agent is supported by development of an anaerobic environment within the liver. In dogs, *C. novyi* is rare and has only been previously reported as a post-mortem diagnosis. In one case, infection was secondary to metastatic pancreatic adenocarcinoma and the other was presumptively diagnosed on histopathology of a hepatic lesion in a dog initially presented for acute collapse.

**Case presentation:**

An 8-year-old spayed, female mixed breed dog was presented for acute onset of hyporexia and vomiting. Serum biochemistry revealed elevated hepatocellular injury and cholestatic liver enzymes. Ultrasound revealed peritoneal fluid accumulation and multiple hepatic masses. Cytologic examination of liver aspirates and peritoneal fluid revealed frequent 4 × 1 μm bacilli with a terminal endospore. Anaerobic bacterial growth isolated from the fluid sample could not be identified using typical laboratory identification techniques. Long-read, whole genome sequencing was performed, and the organism was identified as *Clostridium novyi* type B. Antimicrobial and hepatic support treatment were initiated. The patient re-presented 27 days later, and the follow up liver aspirate with cytology revealed no appreciable bacteria and anaerobic culture was negative. The patient was presented four months later and a large hepatic mass and peritoneal fluid were again identified on abdominal ultrasound. Cytologic examination of the peritoneal fluid revealed bacilli similar to those identified on initial presentation. The patient was euthanized. The most significant finding on necropsy was necrotizing hepatitis with intralesional endospore-forming bacilli compatible with recurrence of *Clostridium novyi* type B. There was no identifiable cause of an anaerobic insult to the liver.

**Conclusions:**

This case demonstrates the diagnostic utility of using cytology as part of the initial diagnostic work up for infectious hepatitis. The cytologic findings coupled with whole genome sequencing and anaerobic culture were crucial for the identification and classification of the organism identified on fine needle aspirate. *Clostridium novyi* type B should be considered when bacilli organisms containing a terminal endospore are identified on liver aspirates collected from canine patients.

## Background

*Clostridium* spp. are Gram-positive, anaerobic, endospore forming bacteria that proliferate when an oxygenated environment becomes anaerobic. Some species, such as *Clostridium piliforme* and *Clostridium perfringens,* cause liver pathology in small animals [1]. *Clostridium novyi* type B is the causative agent of infectious necrotic hepatitis (INH) or black disease, which is most commonly encountered in sheep and cattle. It is rarely identified in other species, including horses and swine. Bacterial endospores in the environment are ingested and reach the liver via portal circulation, where they lay dormant in macrophages until a favorable anaerobic environment develops. This process is commonly associated with migration of liver flukes, including *Fasciola hepatica, Fascioloides magna, or Dicrocoelium dendriticum*, in ruminants [2]*.* In this anaerobic environment, the endospores germinate and proliferate, releasing alpha toxin (TcnA) and beta toxin. The TcnA toxin acts on the actin filament of the cytoskeleton within endothelial cells and the surrounding hepatocytes, leading to cellular detachment, cell death, and leakage of fluid from the damaged endothelial cells [3]. Although this clostridial organism also produces low levels of beta toxin, which causes hemolysis and hepatic necrosis, its role is minimal in the pathogenesis of *Clostridium novyi* type B [3].

There are very few reports in the literature of canine patients being diagnosed with *Clostridium novyi*. Furthermore, those cases that are reported in the literature only identified *Clostridium novyi* as the etiologic agent during postmortem examination and do not describe modern advanced laboratory techniques, such as 16 s rRNA sequencing, matrix-assisted laser desorption/ionization-time-of-flight mass spectrometry (MALDI-TOF,) whole genome sequencing, and polymerase chain reaction (PCR). This case is unique because it highlights the benefits of utilizing fine needle aspiration (FNA) and cytology to better characterize liver lesions identified via diagnostic imaging. To the authors’ knowledge, this is the first report that describes utilizing FNA and cytology to aid in the diagnosis of INH in a canine patient pre-mortem and provides molecular characterization of a *C. novyi* type B isolate associated with necrotizing hepatitis in a dog.

## Case presentation

An 8-year-old female spayed mixed breed dog was presented to the Virginia-Maryland College of Veterinary Medicine Veterinary Teaching Hospital (Blacksburg, VA USA) for acute onset of lethargy, hyporexia, and vomiting. The patient had a one-year history of immune mediated thrombocytopenia (IMTP) that was well controlled with prednisone (1.29 mg/kg/day). Relevant physical exam findings included tachycardia (HR 180 bpm) with a 3/6 left-sided systolic murmur, tachypnea (48 bpm), weak, thready, femoral pulses, 7–8% dehydration, pale mucous membranes with a capillary refill time of 3 s, and a distended abdomen with hepatomegaly and a palpable fluid wave. Blood was collected for complete blood count (CBC) and serum biochemistry (Table 1). Pertinent CBC findings included a mild neutrophilia with a left shift, compatible with infection and/or inflammation. A mild lymphopenia was also present, compatible with acute inflammation and concurrent underlying stress. Pertinent serum biochemistry results revealed markedly increased ALP, markedly increased GGT, and moderately increased ALT. ALT is a marker for hepatocellular injury associated with a plethora of etiologies, including hypoxia, various infectious agents, metabolic derangements, neoplasia, idiosyncratic drug reactions, and toxins, to name a few. ALP and GGT are induction enzymes that increase with cholestasis associated with inflammation, necrosis, lipidosis, or neoplasia. ALP and GGT also increase secondary to induction from drug administration, including corticosteroids in canine patients or phenobarbital. In the present case, hepatocellular injury and cholestasis associated with an unknown etiology were suspected, though some degree of ALP and GGT elevation secondary to prednisone administration was also considered. Other relevant findings include a mild hypertriglyceridemia and a moderate hypochloremia with a markedly increased anion-gap compatible with a mixed acid/base disturbance.

**Table 1:**
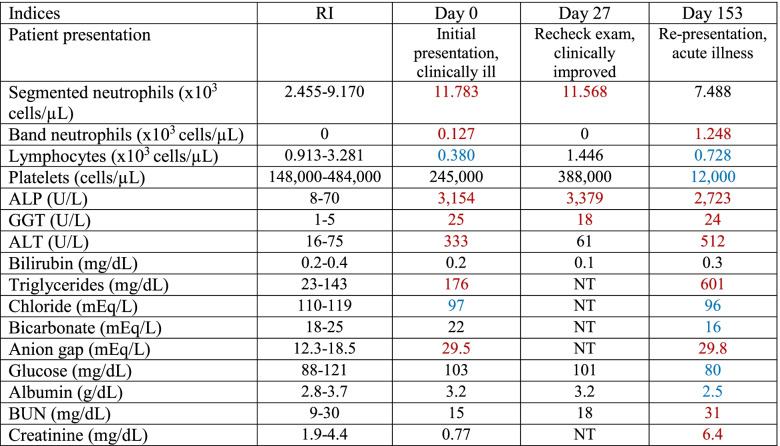
Pertinent hematologic and serum biochemistry values during diagnosis and treatment. Values in red indicate values that are increased outside the reference interval and described within the text. Values in blue indicate values that are decreased outside the reference interval and described within the text

*ALP* Alkaline phosphatase, *ALT* Alanine transaminase, *GGT* Gamma-glutamyl transferase, *RI* Reference interval, *meq/L* Milliequivalent per liter, *mg/dL* Milligram per deciliter, *U/L* Units per liter, *µL* Microliter, *NT* Not tested

**Fig. 1:**
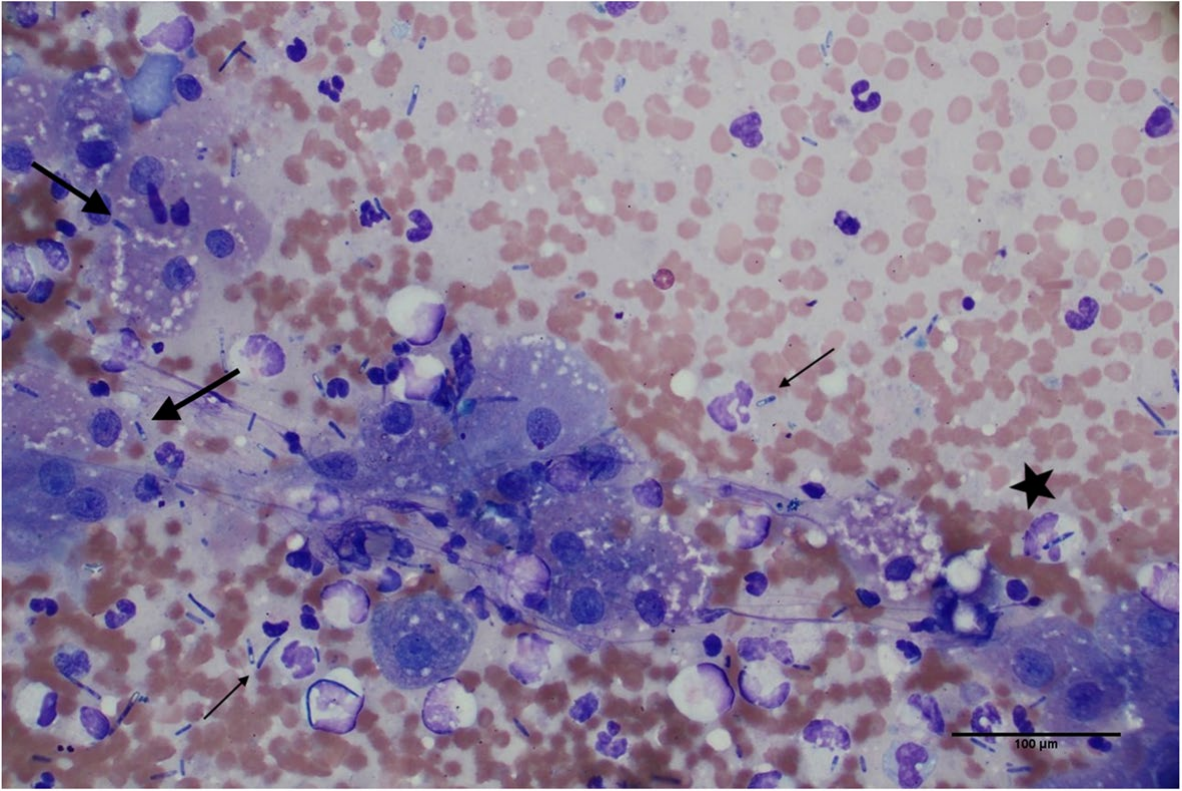
Liver cytology at the time of initial diagnosis (Day 0). There are many vacuolated hepatocytes and moderately increased numbers of degenerate neutrophils admixed with frequent 4 μm × 1 μm bacilli that frequently contain a terminal endospore imparting a “safety pin” morphology (thin arrows). Bacteria are commonly identified within degenerate neutrophils (star) and associated with hepatocytes (thick arrows). Giemsa, 50X magnification

An abdominal ultrasound was performed and revealed multiple hepatic masses and peritoneal fluid. Abdominocentesis was performed and FNA of the liver masses were obtained. Initial cytology of the liver aspirates revealed vacuolar change and evidence of cholestasis. However, due to the clinical suspicion that this was not representative of the lesions noted on ultrasound, a second liver aspirate was performed and resubmitted. The second cytologic sample revealed moderately increased numbers of degenerate and nondegenerate neutrophils, mild hepatocellular atypia, and frequent 1 μm × 4 μm bacilli arranged individually or in short chains. The bacteria frequently contained a terminal endospore imparting a “safety pin” morphology and were commonly found within degenerate neutrophils and associated with hepatocytes (Fig. 1). The peritoneal fluid was classified as a septic exudate with bacilli bacteria that exhibited similar morphology to the bacteria identified in the liver aspirate.

Aerobic cultures were performed on the peritoneal fluid and liver aspirate and revealed no growth of aerobic organisms. Anaerobic culture was not performed on the liver aspirate because the FNA sample was depleted after aerobic culture. Anaerobic culture of the peritoneal fluid revealed 4 + growth of a *Clostridium* species organism that could not be identified on MALDI-TOF as all identification scores were less than 1.5 (Bruker Biotyper, Billerica, MA USA). The organism grew on pre-reduced Brucella agar and phenylethyl alcohol agar (Hardy Diagnostics, Santa Monica, CA USA) in an anaerobic pouch system at 37 °C (AnaeroPouch, Thermo Fisher Scientific, Waltham, MA USA). DNA was extracted from the isolate using a DNeasy Blood and Tissue kit (Qiagen, Valencia, CA USA) following lysis with lysozyme and Triton X-100. The 5’ end of the bacterial 16 s ribosomal DNA gene was amplified using 16 s rRNA primers 4F and 801R [4]. The product was purified using a QIAquick PCR purification kit (Qiagen, Valencia, CA, USA) and sequenced bidirectionally by the Virginia Tech Fralin Life Sciences Genomics Sequencing Center (Blacksburg, VA USA). The 684-base pair organism sequence (OM484269) demonstrated > 99% sequence identity to GenBank accessioned *Clostridium novyi* type B (CP029458.1, AB857215.1), two strains of *C. botulinum* (CP063822.1, CP063816.1), and *C. haemolyticum* (NR_119281.1, NR_024749.1) using NCBI Blast.

Molecular testing to further differentiate between the bacterial species via whole genome sequencing was performed on DNA isolated from the organism. Due to the tendency of this organism to sporulate on solid media, which precluded the isolation of high-quality DNA for sequencing, the organism was suspended in Peptone Yeast Extract Broth with Glucose (PYG, Anaerobe Systems, Morgan Hill, CA USA) and incubated three days at room temperature (approximately 25 °C) under anaerobic conditions. Bacteria were then pelleted by centrifugation for 10 min at 5000 rpm, resuspended in 300 µl sterile water, and processed with garnet beads on a BeadRuptor Elite 24 (Omni International, Kennesaw, GA USA) for 45 s at 6.3 m per second. DNA was extracted according to the QIAamp DNA Mini Kit (Qiagen, Valencia, CA USA) protocol for isolation of genomic DNA from Gram-positive bacteria, using 200 µg/ml lysostaphin (Sigma, St. Louis, MO USA) as the enzymatic lysis solution. Final elution of DNA was in nuclease-free water. The DNA was concentrated by magnetic bead purification with XP AMpure beads (Beckman Coulter, Brea, CA USA), and 1.7 µg of DNA was used for MinION sequencing using the Ligation Sequencing Kit (SQK-LSK109), a FLO-MIN106 flow cell, the Mk1B MinION sequencing device, and MinKNOW software version 19.06.7 (Oxford Nanopore Technologies, Oxford UK). Reads were basecalled using Guppy version 5.0.14. 3 M reads were obtained and the N50 was 1.65 kb. The genome was assembled into a single chromosome with two plasmids using the Minimap2 plugin in Geneious Prime version 2020.2 (Geneious, San Diego, CA USA). The flagellin gene (OM933595) demonstrated 100% sequence identity to *C. novyi* strain 150557 chromosome (CP029458), which is *C. novyi* type B [5]. Additionally, when using the flagellin gene typing scheme described by Sasaki *et al.,* the *C. novyi* type B-specific primer FlanbR bound to the flagellin gene sequence in silico along with the non-specific primer FlaF, with a predicted 427 bp fragment, the size expected for *C. novyi* type B [6]. There was no predicted binding for primers specific for *C. haemolyticum* or *C. novyi* type A [6]. This isolate’s genome had seven glycosyltransferase genes (OM933596, OM933597, OM933598, OM933599, OM933600, OM933601, OM933602) associated with the monoglycosyltransferase activity of *C. novyi* type B alpha toxin; each had 100% sequence identity to glycosyltransferase genes of *C. novyi* type B strain 150557 (CP29458) [5, 7]. Despite the presence of these glycosyltransferase genes, the actual sequence for the alpha toxin gene TcnA was not identified. However, this toxin gene is phage-associated and cannot always be recovered during genome sequencing even in isolates that phenotypically produce alpha toxin [7, 8]. The phospholipase C beta toxin gene (OM933594) was also identified in the isolate described here, with 100% sequence identity to the beta toxin phospholipase C of *C. novyi* type B [7]. Based on the flagellar gene sequence with concurrent evidence of beta toxin and alpha toxin-associated genes, *Clostridium novyi* type B was the final etiologic diagnosis.

The patient was hospitalized overnight and received intravenous fluids, ampicillin-sulbactam (IV, 22 mg/kg every 8 h), enrofloxacin (IV, 10 mg/kg once), maropitant citrate (IV, 0.99 mg/kg once) and dexamethasone (IV, 0.7 mg/kg once). The patient was discharged the next day and treated at home with oral medications for hepatocellular support, pain control, and broad-spectrum antibiotic coverage: gabapentin (4.3 mg/kg PO twice daily as needed), Denamarin (18.2 mg/kg PO once daily for 30 days), maropitant citrate (2.6 mg/kg PO once daily as needed), enrofloxacin (5.8 mg/kg PO once daily for 35 days), and amoxicillin-clavulanic acid (16.1 mg/kg PO twice daily for 35 days). The prednisone dose was reduced to 0.64 mg/kg/day. The patient returned for a recheck examination 27 days later with improved lethargy and resolution of vomiting. At this time, the patient was receiving amoxicillin-clavulanic acid, enrofloxacin, prednisone, and Denamarin. Repeat CBC and serum biochemistry revealed a persistent mild neutrophilia, ALT within reference range, persistently markedly elevated ALP, and mild improvement in GGT and triglyceride concentrations. Ongoing cholestasis with induction from prednisone administration was suspected (Table 1). Repeat abdominal ultrasound revealed a decrease in the size of the liver nodules and increased hepatic echogenicity, compatible with fibrosis. A repeat FNA and cytology of the liver revealed mild vacuolar change, and no cytologic evidence of infectious agents or inflammation. There was no bacterial growth following anaerobic culture of the liver aspirate. The client was instructed to continue amoxicillin-clavulanic acid and enrofloxacin until the dosing regimen was completed (35 days total). Denamarin and prednisone were prescribed indefinitely.

**Fig. 2:**
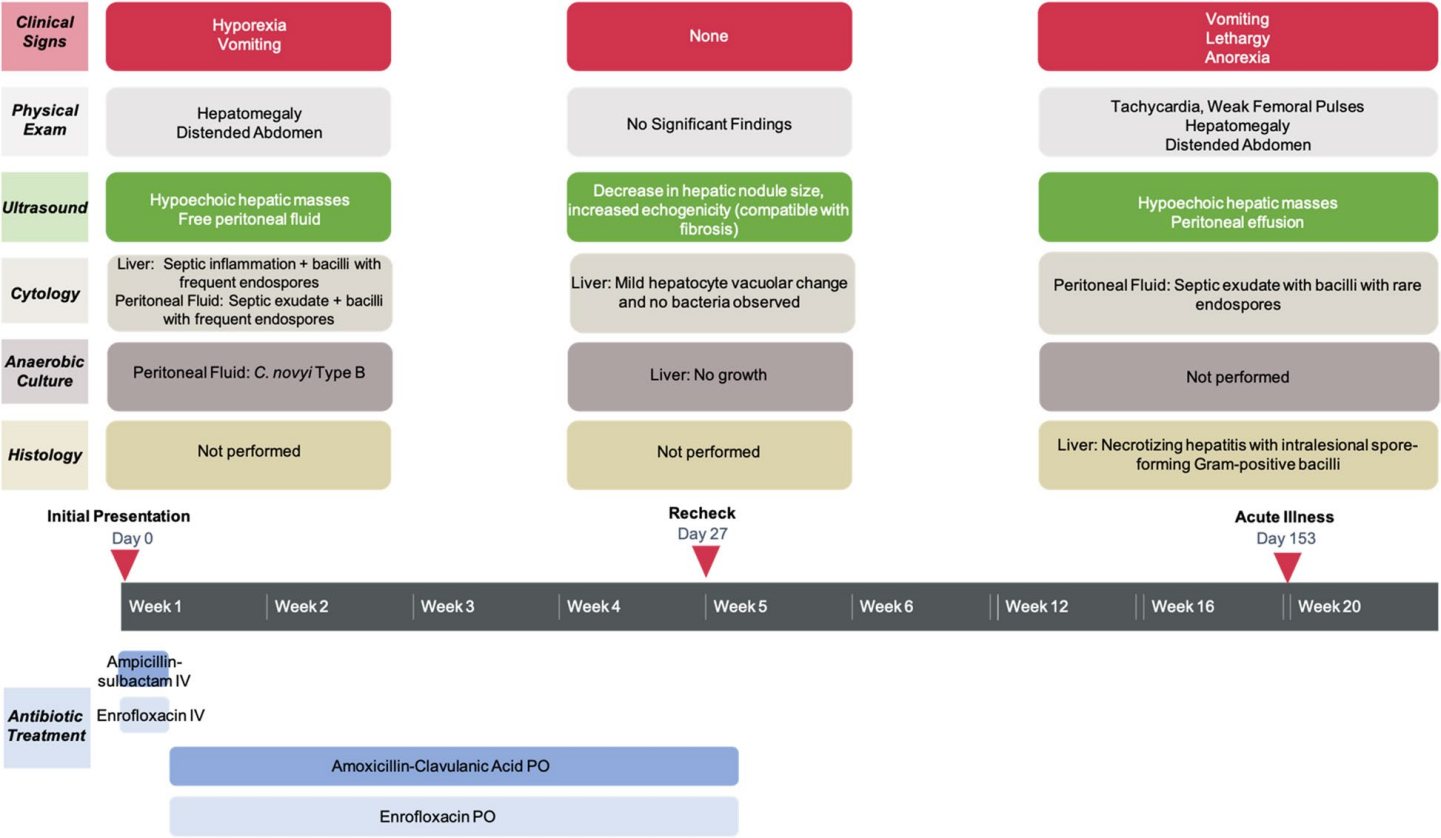
Timeline of clinical data, diagnostic tests, and antimicrobial therapies for this case

**Fig. 3:**
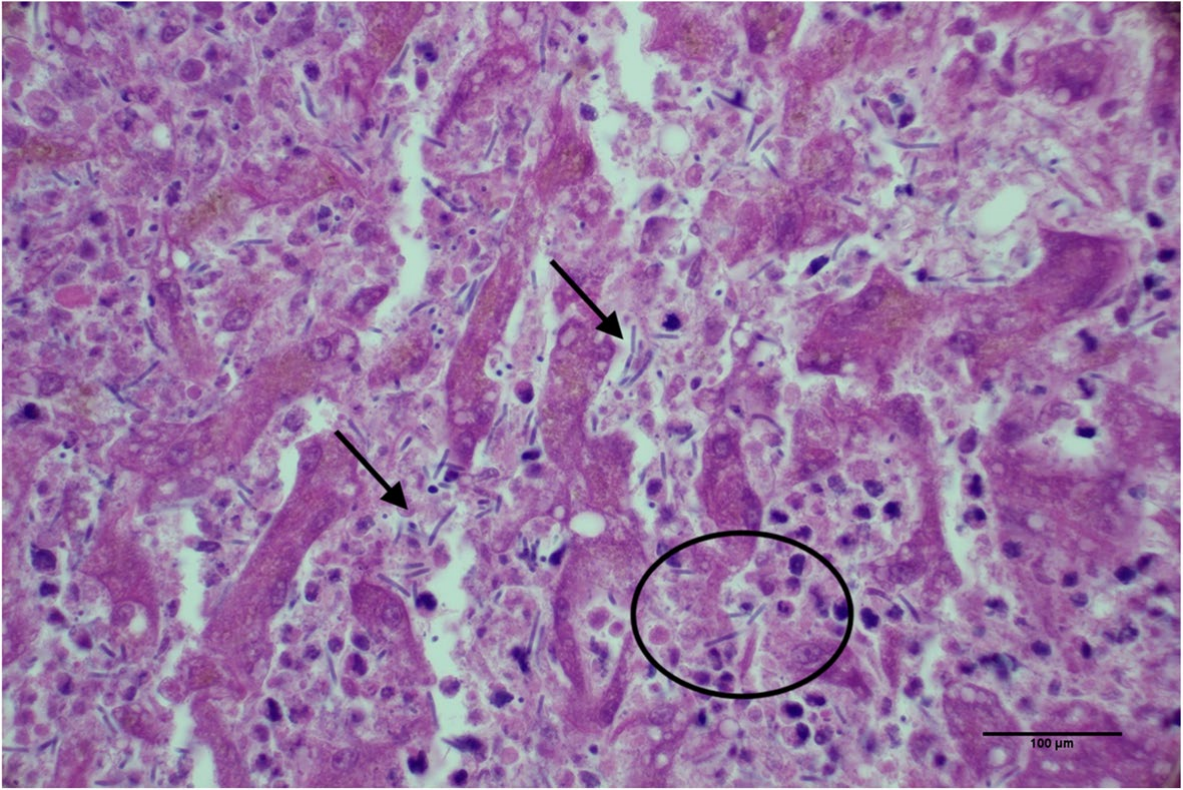
Post-mortem liver histopathology. The tissue architecture is disrupted by regionally extensive foci of coagulative necrosis with inflammation, abundant fibrin (circled), and Gram-positive bacilli measuring up to 20 μm long × 2 μm wide with a terminal endospore (thin arrows). Regions of necrosis are surrounded by bands of degenerate neutrophils and few foamy macrophages. Hematoxylin and eosin, 20X magnification

The patient was discharged but returned four months later for evaluation of acute illness characterized by vomiting, ecchymoses, and petechiae on the ventral abdomen despite ongoing prednisone administration (Fig. 2). CBC revealed marked thrombocytopenia compatible with recurrence of IMTP. A left shift and mild lymphopenia were also noted; these findings were compatible with inflammation and concurrent stress. Pertinent findings on serum biochemistry revealed recurrent hepatocellular injury and cholestasis with potential induction from prednisone administration. The mild hypoglycemia and mild hypoalbuminemia were most compatible with hepatic insufficiency or sepsis with concurrent inflammation. Finally, a mixed acid–base disorder and azotemia of suspected renal origin were also appreciated (Table 1). Abdominal ultrasound revealed a solitary hepatic mass and recurrence of peritoneal effusion. Given the marked thrombocytopenia, the hepatic lesion was not sampled and only the peritoneal fluid was collected for cytologic analysis. The cytologic interpretation was septic exudate and hemorrhagic effusion with rare extracellular and intracellular 4 μm × 1 μm bacilli that rarely contained a terminal endospore, morphologically consistent with the organisms observed five months prior at initial presentation. Given the poor prognosis, the patient was euthanized, and necropsy was performed. Pertinent postmortem findings included identification of a 10 cm mass arising from the medial aspect of the left lateral liver lobe and 1 L of red-tinged peritoneal effusion. The histologic findings of the hepatic mass included regionally extensive foci of coagulative necrosis with inflammation, abundant fibrin, and Gram-positive bacilli measuring up to 20 μm long × 2 μm wide with a terminal endospore (Fig. 3). Regions of necrosis were surrounded by bands of degenerate neutrophils and few foamy macrophages. The final morphologic diagnosis was necrotizing hepatitis with a myriad of intralesional endospore-forming bacilli. Recurrence of *Clostridium novyi* type B was suspected given the patient’s clinical history.

## Discussion and conclusions

*Clostridium novyi* type B is a bacterial organism that commonly affects large animal species including cattle, sheep, and horses. The pathophysiology and ensuing clinicopathologic and histopathologic features have been best characterized in sheep. However, more case reports are emerging in the literature that describe the clinical outcome in other species affected by the disease [7, 9]. In general, the organism is fastidious and can lie dormant in soil for long periods of time and is presumed to be a normal commensal organism of the gastrointestinal flora in large animal species. In these species, spores are ingested with food, at which point the spores travel through the gastrointestinal tract, enter the portal bloodstream, are phagocytosed by macrophages, and lie dormant in the Kupfer cells of the hepatic parenchyma. In anaerobic environments, the organism converts to a vegetative form and begins to proliferate. The most commonly described lesion associated with *Clostridium novyi* type B is secondary to migration of the liver fluke *Fasciola hepatica*, which causes local hepatic necrosis and damage, thus establishing an anerobic microenvironment [3]. However, metabolic derangements associated with lipid accumulation in hepatocytes, abscessation, and toxin exposure can also incite anaerobic conditions [9]. Overall, transition to the active, vegetative stage of the bacteria causes release of alpha and beta toxins. Alpha toxin is the major toxin released by *Clostridium novyi* type B and causes endothelial cell death, edema, and eventual necrosis of the hepatic parenchyma. Sheep are often presented peracutely or are found dead. During the acute presentation, clinical signs are nonspecific, with evidence of inflammation, metabolic acidosis, skeletal muscle enzyme changes, and azotemia [9]. Cattle are often presented with similar clinical signs. Horses exhibit a longer clinical course (12–72 h) and have a grave prognosis. Clinical signs in these animals include icteric sclera, colic, depression, lethargy, nonspecific central nervous system signs, or peritoneal effusion. In an equine case report, a single lesion of the left liver lobe was identified [9]. Overall, the histologic lesions are similar, including coagulative necrosis with infiltration of degenerate neutrophils and large, intralesional, spore-forming bacilli.

This case is unique because *Clostridium novyi* type B has not been previously confirmed to be the primary agent of hepatitis in small animal species. There is a single case report describing the association of *Clostridium novyi* with hepatic abscesses in a dog with concurrent pancreatic acinar carcinoma that had metastasized to the liver and diaphragm [10]. Bacteria were isolated from the liver postmortem but no toxin assays were performed and the isolate was biochemically most similar to *C. novyi* type D. It was hypothesized that the metastatic neoplastic lesions compromised hepatic blood flow, thus creating an anaerobic environment that supported Clostridial proliferation [10]. A second case report describes a German Shepherd dog presented for acute collapse after a presumed traumatic event. A necrotic liver lesion was identified during exploratory laparotomy and the dog was euthanized [11]. Histopathologic examination of the hepatic tissue at necropsy revealed bacilli containing a terminal endospore that was presumed to be *Clostridium novyi* based on histologic appearance but no confirmatory testing was performed [11]. Furthermore, *C. novyi* strain NT has been experimentally administered to dogs as an anti-tumor biologic therapy because of its ability to induce hepatic necrosis and inflammation in necrotic centers of hepatic tumors [12]. *C. novyi* has also been isolated from the liver of an adult dog with no clinical signs of liver disease but that had histologic evidence of interstitial hepatic fibrosis and trabecular rearrangement [13]. This indicates that, as in other animal species, *C. novyi* may lay dormant in the liver of dogs and proliferate only when appropriate anaerobic conditions are present.

It is difficult to differentiate between *C. novyi* and *C. haemolyticum* using typical identification methods such as MALDI-TOF and 16 s rRNA sequencing due to their genetic similarities. *C. haemolyticum (*but not *C. novyi*) is in the Bruker Biotyper database, but it has similar MALDI-TOF spectra to *Clostridium botulinum* groups C and D [14]. *C. botulinum* is not included in the regular Biotyper database, and this may have contributed to the fact that no reliable MALDI-TOF identification could be obtained for the *C. novyi* type B isolated from this patient. Because MALDI-TOF has been widely adopted in many veterinary diagnostic laboratories, it is possible that *C. noyvi* type B has been overlooked in small animal hepatitis and cholangiohepatitis cases due to the difficulty in fully identifying the organism in routine anaerobic cultures. In fact, non-perfringens *Clostridium* spp. are one of the more frequently encountered anaerobic pathogens of the canine hepatobiliary tract, but speciation information is often not reported [15–19]. While 16 s rRNA sequencing assisted with the diagnostic process, it was not possible to differentiate between *C. novyi* and *C. haemolyticum* based on the fragment that was sequenced for this case. Whole genome sequencing finally provided an identification of the organism but processing the isolate was also difficult due to the proclivity of the organism to sporulate, which made DNA extraction for long-read whole genome sequencing a challenge.

Strengths of this investigation include the appropriate use of ultrasound-guided FNA of the liver and successful isolation and molecular typing of *C. novyi* type B. The initial use of clinicopathologic data coupled with ultrasonography and FNA with cytology were noninvasive diagnostic steps at the time of initial presentation. Furthermore, the cytomorphology of the bacilli identified on the liver aspirate was helpful in dictating the subsequent diagnostic steps that were taken, such as utilizing the samples to perform anaerobic culture. Due to the difficulty in identifying the organism and the paucity of information regarding *Clostridium novyi* type B and INH in small animal species, further diagnostic investigation was warranted. The subsequent tests, such as whole genome sequencing, were able to more definitively identify *Clostridium novyi* type B as the etiologic agent in this case.

Weaknesses of this investigation include reliance on FNA and cytology for recheck evaluation of the patient and limited characterization of the bacteria associated with the necrotizing hepatitis lesions at necropsy. At the time of the recheck examination, the serum biochemistry results revealed a persistent mild neutrophilia, which may have been associated with ongoing inflammation in the liver. However, cytology only revealed vacuolar change. Because FNA is only able to collect small samples and few cells from the sampled site, the sample collected at the recheck examination may not have been representative of the whole lesion identified ultrasonographically. This possibility is highlighted by the initial aspirates obtained when the patient was presented to the hospital at the first visit. Therefore, repeat FNA and cytology may have missed ongoing, subclinical infection; however, the lack of growth on repeat culture supported the cytologic findings. Because Clostridial species can lie dormant in lesions not exposed to anaerobic conditions, full resolution of the initial infection is impossible to confirm based on FNA and cytology. Additionally, prednisone is an immunosuppressive drug that can also inhibit the ability to adequately fight off an infection. Although the patient was clinically doing well for several months after cessation of antibiotics, further supporting the presumption that treatment of the infection was successful, the possibility of dormancy within the hepatic parenchyma cannot be ruled out entirely. Furthermore, a cause for recrudescence of thrombocytopenia was never discovered, and the relationship between severe thrombocytopenia and the INH was not fully elucidated. The degree of thrombocytopenia observed is ultimately suggestive of recurrence of IMTP, but consumption due to disseminated intravascular coagulation (DIC) is also possible. If the cause of the thrombocytopenia was truly immune-mediated destruction, this may have been incited by an unidentified rickettsial agent, which can lead to secondary IMTP, vasculitis, and inflammation. Further testing for rickettsial disease would have been a crucial step in the diagnostic work up to further characterize the patient’s clinical signs at re-presentation had euthanasia not been elected. In addition, at the time of re-presentation, although collection of peritoneal fluid was again helpful for identification of bacilli, not all of the clinical signs could be explained by the cytologic appearance of the liver or necropsy findings. Finally, at the time of necropsy the diagnosis of INH caused by *C. novyi* type B was tentatively made based off of the patient history and previous diagnostic work up rather than repeat anaerobic culture or molecular diagnostics.

It is unusual that the INH diagnosis in this case was made pre-mortem. The patient survived five months post-diagnosis with medical management. No cytologic or histopathologic evidence of neoplasia in the liver or other organs was observed and the inciting cause of hepatic anaerobiosis was not apparent on necropsy. It appeared that this patient originally responded well to antimicrobial therapy and supportive care, and there was clinical, ultrasonographic, cytologic, and bacteriologic evidence that the infection resolved within the first month after diagnosis. However, the patient acutely decompensated and the infection recurred four months later. This may have been due to persistence of residual necrotic foci that were resistant to antibiotic penetration. However, it is also possible that there was an additional anaerobic insult on the liver that also allowed proliferation of dormant, sporulated *C. novyi.* It can be speculated that ongoing corticosteroid administration with resultant steroid hepatopathy, underlying inflammation, recrudescence of immune-mediated thrombocytopenia (IMTP), or hypoxic injury may have triggered a second anaerobic insult to the liver. However, marked vacuolar change, vasculitis, hemorrhage, and lesions consistent with inflammation or DIC were not features described on histology.

Overall, identification of *C. novyi* type B in this case was a diagnostic challenge. The organism was not correctly identified by MALDI-TOF and 16 s sequencing did not provide a definitive identification due to the genetic similarity between *C. novyi, C. botulinum*, and *C. haemolyticum*. Furthermore, it was difficult to extract high quality DNA for long-read whole genome sequencing due to the sporulation of the organism. This case also highlights the importance of molecular testing and DNA sequencing to confidently identify the genes that allow differentiation between Clostridial species, such as the flagellin, toxin, and toxin-associated genes.

## Data Availability

Genome sequences described in the study are deposited in GenBank under the accession numbers OM484269 (16 s rRNA) and OM933594-602 (phospholipase C, flagellin, and glycosyltransferase genes). The datasets used and/or analyzed in this study are available from the corresponding author upon reasonable request. Images were captured on a Canon EOS Utility 2, Version 2.14.20.0 by Canon Inc. Tokyo, Japan. Figure 1 resolution is as follows: DPI 1.53 pixels per inch. Figure 2 resolution is as follows: DPI 1.53 pixels per inch.

## Abbreviations

ALP: Alkaline phosphatase
ALT: Alanine transaminase
CBC: Complete blood count
DNA: Deoxyribonucleic acid
FNA: Fine needle aspirate
GGT: Gamma-glutamyl transferase
IMTP: Immune-mediated thrombocytopenia
INH: Infectious necrotic hepatitis
MALDI-TOF: Matrix-assisted laser desorption/ionization-time-of-flight mass spectrometry
RI: Reference interval
rRNA: Ribosomal ribonucleic acid
DIC: Disseminated intravascular coagulation

## References

[1] Center SA. Infectious Diseases of the Liver in Small Animals - Digestive System. Merck Veterinary Manual, Whitehouse Station, NJ: Merck; 2015. Available from: www.merckvetmanual.com/digestive-system/hepatic-disease-in-small-animals/infectious-diseases-of-the-liver-in-small-animals.

[2] Uzal FA, Navarro M. Chapter 23: Infectious Necrotic Hepatitis. In: Uzal FA, Songer JG, Prescott JF Popoff MR, editors. Clostridial Diseases of Animals. Hoboken, NJ: John Wiley & Sons, Inc: 2016. Available from: onlinelibrary.wiley.com/doi/book/10.1002/9781118728291.

[3] Navarro MA, Uzal FA (2020). Pathobiology and diagnosis of clostridial hepatitis in animals. J Vet Diagn Invest.

[4] Persing DH, ed. Molecular Microbiology: Diagnostic Principles and Practice. Third ed. Chapter Two: Application of Identification of Bacteria by DNA Target Sequencing in a Clinical Microbiology Laboratory; Washington, DC: ASM Press; 2016.

[5] Jeong CG, Seo BJ, Kim WI (2016). Diagnosis of sudden death cases during summer season and isolation of *Clostridium novyi*. Korean J Vet Serv.

[6] Sasaki Y, Kojima A, Aoki H, Ogikubo Y, Takikawa N, Tamura Y (2002). Phylogenetic analysis and PCR detection of *Clostridium chauvoei, Clostridium haemolyticum, Clostridium novyi* types A and B, and *Clostridium septicum* based on the flagellin gene. Vet Microbiol.

[7] Jeong CG, Seo BJ, Nazki S, Jung BK, Khatun A, Yang MS, Kim SC, Noh SH, Shin JH, Kim B, Kim WI (2020). Characterization of *Clostridium novyi* isolated from a sow in a sudden death case in Korea. BMC Vet Res.

[8] Eklund MW, Poysky FT, Peterson ME, Meyers JA (1976). Relationship of bacteriophages to alpha toxin production in *Clostridium novyi* types a and B. Infect Immun.

[9] Nyaoke AC, Navarro MA, Beingesser J, Uzal FA (2018). Infectious necrotic hepatitis caused by *Clostridium novyi* type B in a horse: case report and review of the literature. J Vet Diagn Invest.

[10] Love DN, Maddison JE, Finnimore PM, Rothwell TL (1981). Isolation of Clostridium novyi (Cl. oedematiens) from liver lesions in a dog with pancreatic acinar carcinoma. J Small Anim Pract.

[11] Vacher N, Allan P, Sullivan N (2006). Clostridial hepatitis in a dog. Aust Vet Pract.

[12] Krick EL, Sorenmo KU, Rankin SC, Cheong I, Kobrin B, Thornton K, Kinzler KW, Vogelstein B, Zhou S, Diaz LA (2012). Evaluation of *Clostridium novyi*–NT spores in dogs with naturally occurring tumors. Am J Vet Res.

[13] Niza MM, Ferreira AJ, Peleteiro MC, Vilela CL (2004). Bacteriological study of the liver in dogs. J Small Anim Pract.

[14] Library Release Notes, Revision F MBT 8468 MSP. Bruker MBT Compass Library. Billerica, MA: Bruker Daltonics. 2019.

[15] Center SA, Greene CE (2012). Chapter 89: Hepatobiliary Infections. Infectious Diseases of the Dog and Cat.

[16] Jang SS, Breher JE, Dabaco LA, Hirsh DC (1997). Organisms isolated from dogs and cats with anaerobic infections and susceptibility to selected antimicrobial agents. J Am Vet Med Assoc.

[17] Ramery E, Papakonstantinou S, Pinilla M, McAllister H, Jahns H, Gallagher B, O'Brien PJ (2012). Bacterial cholangiohepatitis in a dog. Can Vet J.

[18] Wagner KA, Hartmann FA, Trepanier LA (2007). Bacterial culture results from liver, gallbladder, or bile in 248 dogs and cats evaluated for hepatobiliary disease: 1998–2003. J Vet Intern Med.

[19] O'Neill EJ, Day MJ, Hall EJ, Holden DJ, Murphy KF, Barr FJ, Pearson GR (2006). Bacterial cholangitis/cholangiohepatitis with or without concurrent cholecystitis in four dogs. J Small Anim Pract.

